# Editorial: Computational modeling and machine learning methods in neurodevelopment and neurodegeneration: from basic research to clinical applications

**DOI:** 10.3389/fncom.2024.1514220

**Published:** 2024-11-08

**Authors:** Noemi Montobbio, Roberto Maffulli, Anees Abrol, Pablo Martínez-Cañada

**Affiliations:** ^1^Department of Health Sciences (DISSAL), University of Genoa, Genoa, Italy; ^2^EXUS AI Labs, London, United Kingdom; ^3^Center for Translational Research in Neuroimaging and Data Science, Georgia State University, Atlanta, GA, United States; ^4^Research Centre for Information and Communications Technologies (CITIC), University of Granada, Granada, Spain; ^5^Department of Computer Engineering, Automation and Robotics, University of Granada, Granada, Spain

**Keywords:** computational modeling, machine learning, neurodevelopmental disorders, neurodegeneration, clinical

Computational models and machine-learning methods are increasingly valuable for understanding how neural networks in the brain process information, and how this information influences decision-making and behavior. Abnormalities in these networks are linked to various brain disorders. Advances in brain simulation, machine learning, and neuroimaging have helped bridge different brain scales and uncover the processes underlying cognitive, motor and behavioral impairment in neurodevelopmental and neurodegenerative disorders.

The effective application of computational approaches still faces several challenges, including: the multiple spatial scales involved; the issue of interpretability of machine learning models, hampering transferability to clinical practice; and the lack of robust validation of non-invasive biomarkers of neural disorders. These challenges motivated us to edit the Research Topic “*Computational Modeling and Machine Learning Methods in Neurodevelopment and Neurodegeneration: from Basic Research to Clinical Applications*”, culminating with the acceptance of 10 insightful papers that explore the subject from diverse perspectives using various innovative tools.

The contributions covered a variety of themes, including disease diagnosis (Ruppert-Junk et al., Turrisi et al., Fernández-Ruiz et al.), disease subtype or stage classification (Chen et al., Zheng et al.), predictors of disease progression (Zhang et al.), brain network simulation (Monteverdi et al., Moore et al.), lesion segmentation (Zaman et al.), and clustering methods in medicine (Poulakis and Westman). Deep learning was widely present, and was explored from different perspectives, from the proposal of novel model architectures (Zaman et al.) to an analysis of validation and reproducibility issues (Turrisi et al.). Although most papers focused on MRI data, other neuroimaging modalities such as EEG (Zheng et al.) and speech (Fernández-Ruiz et al.) were explored as well.

Computer-aided diagnosis, as well as disease subtype or stage classification, are among the most frequently addressed tasks in machine learning studies in healthcare (Chan et al., 2020). In the present Research Topic, the study by Ruppert-Junk et al. investigated the use of [^18^F]-FDG PET imaging to diagnose Parkinson's disease (PD) by focusing on midbrain metabolism, particularly in the substantia nigra. A machine learning model using random forest classification achieved high sensitivity and accuracy in distinguishing PD patients from healthy controls. Fernández-Ruiz et al. introduced a non-invasive method for identifying Smith–Magenis syndrome using machine learning techniques, focusing on cepstral peak prominence (CPP) from voice samples. The study significantly contributes to the theme of using computational methods for neurodevelopmental conditions by offering a potential clinical application for early diagnosis? Chen et al. explored the use of graph-based convolutional networks (GCNs) to classify multiple sclerosis (MS) clinical forms based on brain morphological connectivity from T1-weighted MRI data. The authors show how the approach outperforms state-of-the-art 3D Convolutional Neural Networks (CNNs) methods, offering insights into how computational models can help differentiate between MS subtypes.? Zheng et al. proposed a novel framework for epilepsy diagnosis using a complexity-based Graph Convolutional Neural Network (GCNN) to analyze multi-channel EEG signals across normal, acute, and chronic stages. By incorporating five complexity measures, their model achieved high accuracy in distinguishing between these phases, thus highlighting its potential in detecting chronic epilepsy for more effective intervention. Zhang et al. studied gray and white matter alterations in children affected by sensorineural hearing loss (SNHL) based on their auditory brainstem response. They identified independent predictive factors to study SNHL progression in children, highlighting the value of quantitative T1 assessments in specific regions of interest and tracking white matter and myelin volume and fraction parameters.

Automatic medical image segmentation tools are highly required by the medical community, and several deep learning techniques have been successfully applied in this field in recent years (Ramesh et al., 2021). Zaman et al. presented the Adaptive Feature Medical Segmentation Network (AFMS-Net) for 3D brain lesion segmentation. The network uses novel encoder-decoder structures for high-performance, computationally efficient segmentation, significantly advancing clinical imaging applications in scenarios requiring quick and efficient identification of key lesion areas.

Computational simulations of brain network alterations linked to neurological diseases can be a powerful and cost-effective tool to indicate new directions in clinical research (D'Angelo and Jirsa, 2022). Monteverdi et al. employed multiscale brain modeling using The Virtual Brain (TVB) with MRI data to simulate brain networks in patients with Alzheimer's disease (AD) and frontotemporal dementia (FTD). Their simulations revealed distinct disease-specific alterations in connectivity and synaptic transmission for each condition, which correlated with individual clinical profiles. These insights enhance our understanding of dementia mechanisms and may guide the development of personalized therapeutic strategies. Moore et al. proposed a novel deep learning approach to model neurodegeneration in the visual cortex through progressive lesioning of a convolutional neural network, also including a mechanism to simulate neuroplasticity by allowing the model to adapt to new information even after sustaining simulated damage. The authors show that incorporating neuroplasticity resulted in a smoother and slower decline in model performance, aligning with observed disease-related cognitive decline patterns. Overall, findings suggest that integrating neuroplasticity into deep learning models could enhance disease understanding and support testing rehabilitation approaches.

Finally, the issue of validation and reproducibility of computational techniques is raising growing interest (McDermott et al., 2021). Poulakis and Westman contributed with a letter elaborating on the applications and challenges of clustering for studying heterogeneity in psychiatric and neurological disorders. They emphasized the importance of careful methodological selection, validation, and expert involvement to address the limitations and improve the interpretation of clustering results in high-dimensional datasets. Turrisi et al. highlighted the importance of adhering to shared guidelines to ensure the reliability, robustness, and reproducibility of ML in healthcare. Using the challenging problem of Alzheimer's disease detection from MRI scans as a case study, the authors demonstrated best practices in data handling, model design, and assessment, while also revealing the susceptibility of prediction accuracy to modeling choices.

We believe that this Research Topic will provide readers with a stimulating overview of current themes in computational modeling and machine learning as applied to neurodevelopment and neurodegeneration. The contributions emphasize both the potential and the challenges of these approaches, offering insights that can inspire future research and ultimately support clinical advancements in diagnosing and treating brain disorders.
